# Cognitive Effects of Taurine and Related Sulphur-Containing Amino Acids: A Systematic Review of Human Trials and Considerations for Plant-Based Dietary Transitions

**DOI:** 10.3390/foods15040634

**Published:** 2026-02-10

**Authors:** Jack A. Moore, Alecia L. Cousins, Rebecca M. J. Taylor, Amy R. Griffiths, Hayley A. Young

**Affiliations:** School of Psychology, Faculty of Medicine, Health and Life Science, Swansea University, Swansea SA2 8PP, UKa.l.cousins@swansea.ac.uk (A.L.C.); a.r.griffiths@swansea.ac.uk (A.R.G.)

**Keywords:** taurine, sulphur-containing amino acids, cognitive function, mood, plant-based diets, sustainable diets

## Abstract

As diets shift towards more plant-based patterns, nutrients mainly supplied by animal-sourced foods are receiving greater attention. Among these are sulphur-containing amino acids (SCAAs) such as taurine, methionine, and cysteine. These compounds play important roles in neuroprotection, antioxidant defence, and cellular signalling; functions that are closely linked to cognitive health. This systematic review examined the effects of SCAA supplementation on cognitive performance in randomised controlled trials (RCTs). Eight RCTs involving 244 healthy participants met the inclusion criteria. All trials focused exclusively on taurine; no studies were found that tested methionine or cysteine. Each used an acute, single-dose design, assessing key cognitive domains and mood outcomes. Overall, acute doses of taurine (typically 1–3 g, up to ~50 mg/kg) produced, at best, small and inconsistent improvements in cognitive function. Most cognitive outcomes showed no effect. Trials that combined taurine with caffeine showed more reliable performance benefits, but they did not isolate taurine’s independent effects. Similarly, any positive effects on mood or well-being were minor, inconsistent, and typically observed only under specific conditions, such as when taurine was combined with caffeine, exercise, or sleep deprivation. Importantly, none of the studies measured participants’ habitual diets, baseline SCAA status, or specifically recruited individuals with low intake of animal-source foods. This means the cognitive effects of reduced SCAA intake in plant-based diets remain unknown. Current evidence from acute taurine trials provides limited support for short-term benefits to cognition or mood. Longer-term, well-designed studies are urgently needed. These should assess habitual diet and baseline SCAA status and focus on populations with lower animal-derived food intake. Only then can we determine whether lower SCAA availability in plant-based diets represents a nutritional ‘green gap’ with implications for brain health.

## 1. Introduction

Climate change is a significant global challenge facing society. Broadly defined as the alteration in temperature and weather patterns, climate change occurs as a result of human activity either directly or indirectly [1]. If not addressed, climate change will have significant consequences for global temperatures, weather, and flooding [2], with further negative impacts on health [3,4], food security [5], and water supplies. Greenhouse gas emissions are the key driver of climate change, with approximately a third of all greenhouse gas emissions coming from the food system, e.g., methane produced by cattle digestion, nitrous oxide used to fertilise crops [6]. Consequently, shifting populations to more sustainable diets is high on the global sustainability agenda [7]. The EAT-Lancet commission has recommended a healthy and sustainable reference diet, largely consisting of plant-based foods, small amounts of dairy, and very little meat [8,9]. However, shifting dietary patterns to reduce or remove meat consumption may have unintended consequences for cognition, potentially creating a nutritional ‘green-gap’ by compromising the intake of nutrients with key roles in supporting neuroprotection and cognition [10,11,12,13]. Additionally, multiple studies have indicated that following and adapting to plant-based diets may result in significantly lower plasma sulphur-containing amino acid (SCAA) levels [14,15].

SCAAs, namely methionine (MET), cysteine (CYS), and taurine (TAU), are amino acids crucial for maintenance of cellular function and health [16]. MET and CYS are incorporated into proteins and contribute to the synthesis of TAU [17]. While TAU is not incorporated into proteins, it has physiological significance due to anti-inflammatory properties [18] and its role in regulating blood pressure [19] and insulin sensitivity [20]. Specifically, TAU has garnered increased attention due to its potential neuroprotective properties and influence on cognitive function [21,22,23]. In animal models, supplementing TAU led to improvements in memory [24,25]. Additionally in mice, mood disorders such as depression were associated with TAU levels in the extracellular fluid of the medial frontal cortex, where TAU concentration was lower in mice exhibiting depressive symptoms [26,27]. A recent study also demonstrated that TAU may be neuroprotective, with lower blood plasma levels of TAU being associated with higher dementia risk level [28]. As such, evidence suggests that TAU, and potentially SCAAs more broadly have importance for cognitive health. While CYS and TAU can be synthesised endogenously [29], the highest concentrations of SCAAs in humans are obtained through animal-based dietary sources [17] including meat, dairy products and fish. Therefore, it is important to consider that shifting dietary patterns towards sustainable, plant-based diets may inadvertently reduce SCAA intake with potential consequences for cognitive health.

To date, most research on the cognitive effects of SCAAs has either focused on (1) supplementation in clinical populations with neurodegenerative conditions—leaving the long-term effects in healthy adults largely unexplored [30]—or (2) acute supplementation, often involving taurine in energy drinks at supradietary doses far exceeding typical daily intake (estimated at 40–400 mg) [31]. Notably, rather than solely representing a limitation, these supradietary levels may be necessary to elicit acute neurocognitive effects, for instance by achieving the concentrations required to activate specific inhibitory receptors (e.g., GABA-A) that are less sensitive to lower physiological levels [32]. Crucially, a distinction must be drawn between the effects of supplementation—aimed at optimising performance in replete individuals—and the issue of dietary depletion, where the goal is restoring homeostatic levels to prevent decline. While the transition to plant-based diets raises specific concerns regarding the latter, current research largely assesses the former, creating a disconnect between the available evidence and the emerging public health concern.

Therefore, this review was designed to address two primary research questions: (1) Does SCAA supplementation have measurable acute effects on cognitive function and mood? and (2) Is there evidence regarding the cognitive effects of SCAA restoration in the context of low dietary intake? To answer these questions, this review focuses on randomised controlled trials (RCTs) that measure cognitive outcomes (e.g., memory, attention, and executive function), and mood (e.g., anxiety, depression, and emotional well-being). Additionally, it aims to identify which dosages, if any, yield the most substantial benefits. Overall, this review aims to clarify current evidence for SCAAs as cognitive modulators and highlight the critical knowledge gaps that must be addressed to understand the cognitive implications of the global shift toward sustainable diets.

## 2. Materials and Methods

This systematic review was conducted following the Preferred Reporting Items for Systematic Reviews and Meta-Analyses (PRISMA) guidelines [33] to ensure methodological transparency and reproducibility. The review was developed using the Population, Intervention, Comparison, Outcomes, Context (PICOC) framework, which is an extension of the PICO (Population, Intervention, Comparison, Outcomes) framework [34]. This framework guided the exploration of SCAA efficacy (Intervention) versus placebo control (Comparison) on cognition and mood (Outcomes) in healthy adults (Population), within the contexts of both dietary reduction and varying supplementation states (Context). The review protocol was preregistered with PROSPERO (ID: CRD42024574453). The completed PRISMA 2020 checklist is provided in the Supplementary Materials (Table S1).

### 2.1. Search Strategy

An initial comprehensive search was carried out across PubMed, EMBASE, and PsycINFO to identify RCTs published in English that investigate the effects of SCAAs on cognition and mood. A manual search of reference lists from relevant articles was conducted to identify additional studies. This search was conducted between July and August 2024 and included all studies up to September 2024. A further update search was conducted on 1 September 2025. Key words for the database searches were identified from the existing literature and the search strategy used variations of the key words in conjunction with Boolean operators (AND/OR) to account for differences in spelling. The key words included sulphur-containing amino acids, sulphuric amino acid, methionine, cysteine, taurine, cognition, memory, attention, executive function, mental performance, neurocognitive, cognitive score, brain function, information processing, reaction time, mood, depression, anxiety, and energy.

### 2.2. Eligibility Criteria

The PICOC framework guided the development of the inclusion and exclusion criteria (Table 1) with additional considerations applied relating to type of publication and publication language.

### 2.3. Screening and Data Extraction

Two independent reviewers (JM and RT) performed the initial screening of titles and abstracts to identify potentially relevant studies. Full-text articles were then retrieved and assessed against the eligibility criteria by both reviewers. A third reviewer (HY) acted as an arbitrator in cases of disagreement. To ensure fidelity, reviewers met to discuss any disagreements, and conflicts were resolved through discussion until full agreement was reached.

The review team developed a data extraction template to ensure comprehensive and systematic extraction of study characteristics. The extracted data included publication details, participant characteristics, study design, intervention details, cognitive and mood outcomes, results, and conclusions. Where necessary, the authors of studies were contacted to provide missing or unclear data.

### 2.4. Risk-of-Bias Assessment

The risk of bias for each study was assessed independently by reviewers JM and RT using the Cochrane Risk of Bias 2 Tool [35]. Any disagreements were resolved through discussion. The following sources of bias were assessed: randomisation process, deviations from intended interventions, missing outcome data, measurement of the outcome, and selection of the reported result. Studies were classified as having either low risk of bias, some concerns of bias, or high risk of bias. Across all studies, there were no classifications for high risk of bias, with most sources classed as having low risk of bias. Four of the studies were marked as having some concerns of bias in the selection of the reported results. Therefore, the sources’ reported results were carefully examined to make interpretations for the present study (Table 2).

### 2.5. Data Synthesis

A narrative synthesis was performed to summarise the characteristics and findings of the included studies. A meta-analysis was not suitable due to the complex differences in population outcomes, dosages, and combinations with differing active ingredients. This narrative synthesis included tabulated data that described each study’s design, intervention details, cognitive and mood measures, and main findings and conclusions. The narrative synthesis identified key trends, inconsistencies, and gaps in the literature regarding the effects of sulphur-containing amino acids on cognition and mood.

## 3. Results

### 3.1. Search Results

**Figure 1:**
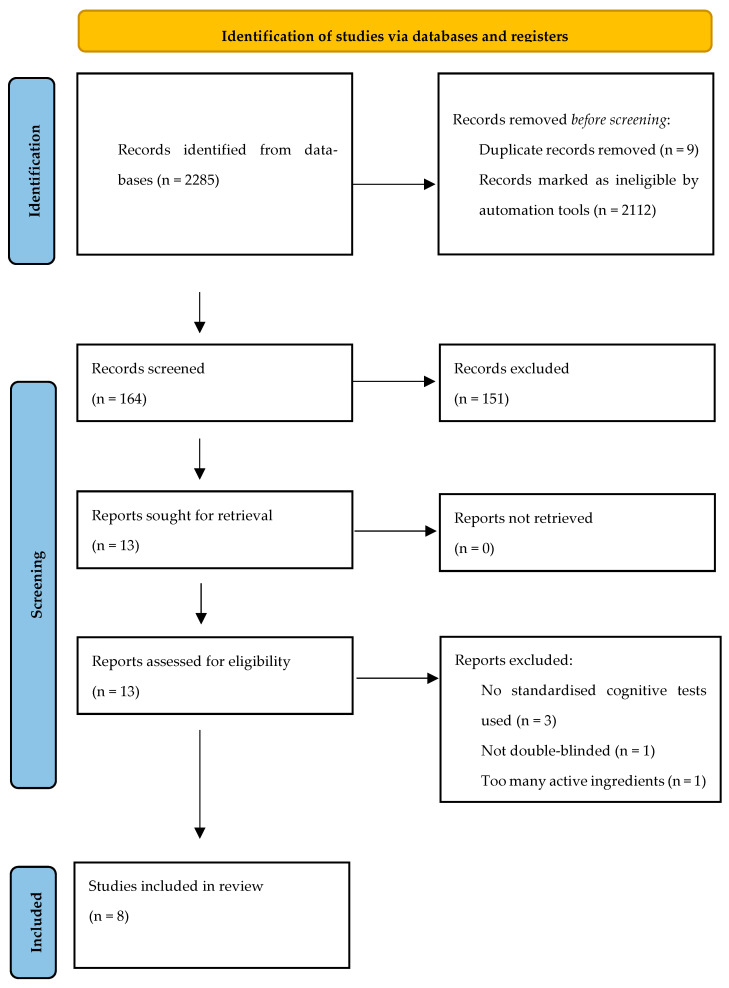
PRISMA flow chart of the screening process.

The PRISMA flow diagram (Figure 1) summarises the study selection process. Database searches identified 2285 records. After removal of duplicates (*n* = 9) and records removed prior to screening (*n* = 2112), 164 records were screened by title and abstract, of which 151 were excluded. Thirteen full-text reports were sought and assessed for eligibility, and five were excluded (no standardised cognitive tests used, *n* = 3; not double-blinded, *n* = 1; too many active ingredients, *n* = 1). Eight studies were included following the search.

### 3.2. Included Studies

While all included studies utilised a validated cognitive test, mood was not assessed in every trial. Across all eligible RCTs, TAU was the sole SCAA investigated; no studies utilising MET or CYS met the inclusion criteria. Crucially, regarding the review’s first objective, no studies were found that assessed cognitive outcomes in the context of dietary depletion or SCAA restoration. Consequently, all eight included studies investigated the acute effects of supplementation in healthy populations (Table 3).

### 3.3. Taurine and Cognitive Function

All eight studies were RCTs assessing cognition through standardised tests. The total study population was as follows: *N* = 244 (range: 10–80), 109 female, 135 male, aged 18–45. Cognitive domains assessed were attention (selective, sustained, and divided attention), executive function (cognitive control and decision-making), memory (working memory and immediate recall), perceptual processing, psychomotor performance, reaction time, and motor control. The findings in relation to the effects of TAU on each of these domains are outlined below.

#### 3.3.1. Attention

Attention was a cognitive domain explored across two studies. Giles et al. [38] used the Attention Network Test to investigate the effects of TAU on alerting, orienting, and executive control components of attention. TAU administered alone showed no significant effect on attention. However, when combined with glucose, there was a significant improvement in attentional control, particularly in tasks requiring orienting attention, indicating faster and more efficient shifting of attentional focus. This pattern suggests that TAU does not globally increase alertness but may modulate specific attentional subsystems under conditions of metabolic support. Notably, no consistent effects were observed on the alerting network, which is closely linked to noradrenergic arousal, reinforcing the view that TAU’s attentional effects are subtle and domain-specific rather than stimulant-like.

Seidl et al. [36] utilised the D2 test of attention to measure selective and sustained attention in a sample of graduate students. The findings demonstrated that a combination of caffeine (CAF) (80 mg), TAU (1 g), and glucuronolactone (600 mg) significantly improved sustained attention scores, although the independent effects of TAU alone were not explored. From the limited available data, TAU alone does not appear to significantly improve attention but may work synergistically with other active compounds to enhance attention, particularly during complex, attention-demanding tasks.

Mechanistically, these limited findings are consistent with TAU’s known neuromodulator role within inhibitory neurotransmitter systems. TAU acts as an agonist at both glycine and extra-synaptic GABA-A receptors, which are centrally involved in regulating cortical excitability, signal-to-noise ratio, and attentional stability.

#### 3.3.2. Executive Function

Three studies explored the influence of TAU on executive function, e.g., cognitive control, inhibition, and decision-making. Ozan et al. [42] explored the effect of TAU on cognitive control via the Stroop test in elite athletes, finding that 3 g of TAU alone significantly improved accuracy and reaction times in incongruent Stroop trials compared to placebo, indicating a selective benefit of TAU supplementation. This selective effect on incongruent trials suggests a potential influence on inhibitory control and conflict monitoring rather than general processing speed. Interestingly, those who received the combination of TAU and CAF showed improved performance across all Stroop trials, indicating a general effect of enhanced cognitive control and faster, accurate responses. Liu and Rong [43] challenged these claims with contrasting findings which demonstrated CAF to be the driver of improved performance during the Stroop task. In their repeated sprint exercise paradigm conducted under hypoxic conditions, they found CAF to significantly improve reaction time for incongruent trials compared to the combination of TAU and CAF, but not for congruent trials. The authors found no evidence to suggest that TAU, on its own, influences cognitive control, even in incongruent trials. Additionally, Lassiter et al. [39] explored the impact of TAU-containing beverages on executive function. The intervention (160 mg CAF, 2 g TAU, 400 mg Panax ginseng) did not produce specific effects on performance; improvements in Go/No Go tests and Stroop reaction times were observed, but as these occurred in both placebo and active groups, they were attributed to exercise rather than the intervention. Notably, this study assessed executive performance both before and after prolonged cycling, providing an exercise-fatigue context distinct from the other trials. Overall, the evidence concerning the effects of TAU-based interventions for executive function is mixed; while TAU appeared to selectively enhance inhibitory control in incongruent Stroop trials in some findings (faster reaction times with fewer errors [42]), this finding was not supported by other studies using the same task [43]. Where consistent enhancements were observed, this was largely attributable to combination interventions where CAF was present.

#### 3.3.3. Working Memory and Immediate Recall

Working memory, particularly the ability to temporarily hold and process information, is crucial for complex cognitive tasks. This was examined in two studies. Giles et al. [38] used the N-back task to assess working memory performance in habitual CAF users. TAU only reduced response time in the verbal N-back in the 1-Back condition in the absence of CAF. Response time was also lower in the object 3-Back condition compared to placebo. Ultimately, this suggests that TAU significantly improves participants’ working memory for both low and high cognitive load. More precisely, in a verbal N-back, this reflects working memory maintenance/updating efficiency rather than episodic ‘recall’, whereby faster responses without a loss of accuracy indicate more efficient processing. Furthermore, there was no antagonistic effect of TAU and CAF recorded for working memory. Importantly, accuracy was preserved across conditions, indicating that observed benefits reflected processing efficiency rather than speed–accuracy trade-offs. A second study [41] explored effects of three different commercially available energy drinks containing active ingredients on working memory, operationalised using the N-back task. In contrast to the findings of Giles, no influence of TAU was observed. The results demonstrated a significant improvement in working memory for those who were in condition A (CAF and glucose, no TAU) compared to those in the control condition. When TAU was combined with CAF and glucose, this significant effect diminished, suggesting an antagonistic effect from TAU.

With regards to immediate memory recall performance, Alford et al. [37] found that short-term memory performance improved after supplying their participants with a commercially available energy drink. The findings demonstrated that participants who received the combination of TAU (1 g), CAF (80 mg), glucose (5.25 g), and glucuronolactone (600 mg) in a 250 mL beverage demonstrated significantly improved immediate recall memory across three different controlled studies compared to those who received either a placebo ‘energy drink’, sparkling water, water, or no drink. The recall task involved rapid encoding and immediate free recall of word lists, capturing short-term retention rather than long-term memory formation. However, as TAU was only administered in combination with other active compounds, it is difficult to draw conclusions on its effects on immediate memory recall. In summary, while TAU shows potential for improving performance in memory tasks, study methodologies and findings are heterogeneous, and the effects of TAU appear context-dependent, varying across task complexities and ingredient interactions. Across both working memory and immediate recall paradigms, any apparent benefits were either modest or contingent on co-administration with other bioactive ingredients.

#### 3.3.4. Reaction Time

Several studies examined the effect of TAU on reaction time using a range of cognitive paradigms including the Stroop test, visual oddball task, stimulus degradation task, reaction time task, and choice reaction time. When administered in isolation, the effects of TAU were inconsistent. Two studies found that supplementation with TAU alone did not enhance reaction time across any of the reaction time paradigms [40,43]. In one study [38], TAU significantly improved reaction times in the N-back task, indicating superior performance vs. placebo. This effect emerged within a working memory paradigm rather than a simple sensorimotor task, suggesting possible domain specificity. The findings suggest task-specific effects where benefits may be more pronounced where cognitive control is required.

Performance during reaction time tasks often improved following a combination of CAF and TAU. Using the Stroop Test, Ozan et al. [42] examined reaction times in elite athletes under anaerobic and aerobic stress. The combination of CAF and TAU significantly improved both reaction times and accuracy, demonstrating their synergistic effect even under physically taxing conditions. Similarly, Alford et al. [37] studied the effects of CAF and TAU on reaction time using the five-choice reaction time task. Participants who received a combination of CAF and TAU demonstrated a significant reduction in reaction times. In contrast, findings from three studies suggest CAF to be the active ingredient that elicits benefits to reaction time. Liu and Rong [43] found that reaction time was reduced during the Stroop task for those who received a combination of TAU with CAF, although CAF alone appeared to have the clearest benefit for reaction times. Peacock et al. [40] assessed reaction times using both the visual oddball task and the stimulus degradation task. The findings demonstrated a limited effect of TAU, but CAF alone significantly improved performance under degraded visual conditions. Furthermore, Giles et al. [38] also found that CAF alone significantly improved reaction time across all tasks, further supporting CAF’s robust facilitatory effect.

Lassiter et al. [39] further illustrate how task context and additional components can influence outcomes. In a sample of healthy trained cyclists, participants completed the choice reaction time task after consumption of a carbonated energy drink. No differences were found between placebo and the active drink containing CAF, TAU, and Panax ginseng. The authors reported that faster reaction times were observed after exercise in both the active and placebo groups. Overall, the findings show that the effects of TAU on reaction time are context- and task-dependent, with evidence suggesting further synergistic effects of administration of TAU in combination with other active ingredients. Across studies, simple reaction time tasks were least sensitive to TAU, whereas paradigms embedding attentional or working memory demands were more likely to show isolated effects.

### 3.4. Taurine and Mood

Four studies assessed mood in their RCTs [36,37,38,41]. The total study population was as follows: *N* = 174 (n range: 10–80), 83 female, 91 male, aged 18–35. Only one study investigated the effects of TAU in isolation from other active ingredients [38]. All other studies used combinations of active components. Aspects of mood assessed across studies included alertness, fatigue, well-being, and anxiety.

Giles et al. [38] used the Profile of Mood States and a Caffeine Withdrawal Questionnaire to evaluate mood states in habitual CAF consumers. There was a significant effect of TAU on fatigue, but lowered feelings of vigour in the absence of CAF, and it was associated with heightened CAF withdrawal symptoms. In contrast, CAF improved vigour, reduced fatigue, and alleviated withdrawal symptoms such as headaches. The combination of TAU and CAF generally mirrored the results from CAF; however, one interaction showed increased vigour over time. Overall, the findings indicate that TAU’s influence on mood was inconsistent and context-dependent, in contrast to the more robust and predictable effects of CAF and its combination with TAU.

Alford et al. [37] measured subjective alertness using a 100 mm VAS scale after participants consumed Red Bull. Following supplementation, subjective ratings of alertness significantly increased compared to placebo, while fatigue ratings declined. This suggests that the combination of CAF and TAU contributes to increased energy and alertness levels.

Seidl et al. [36] used the Basler Befindlichkeitsbogen Questionnaire to evaluate the effects of CAF, TAU, and glucuronolactone on well-being. Participants in the placebo group showed significant declines in well-being, vitality, and social extroversion over the session, whereas those in the active intervention group did not. These findings suggest that the combination may help maintain mood and well-being, particularly during periods of fatigue such as late at night. One study specifically examined the effects of energy drink consumption on anxiety using the State–Trait Anxiety Inventory (STAI).

García et al. [41] conducted a study on medical students to determine the acute effects of energy drinks on mood, including state anxiety, using the STAI. The study divided participants into groups consuming different energy drinks (labelled as A, B, and C) and a control group that consumed carbonated water. Drink A contained 149.5 mg CAF and 23 g of glucose. Drink B contained 147.2 mg CAF, 49.6 g glucose, and 1.84 g of TAU. Drink C contained 155 mg CAF, 52.8 g of glucose, and 1.95 g of TAU. The results indicated that Drink C significantly reduced STAI scores post-consumption, suggesting that Drink C was responsible for significantly reducing anxiety. This reduction implies that some active ingredients may have calming effects, potentially counteracting the stimulating effects of CAF through the interaction with other ingredients. However, the comparative analysis across all groups showed no significant differences in the percent change in STAI scores, indicating that while Drink C had a notable impact on reducing state anxiety, the effect was not uniformly observed with the other drink conditions. Overall, evidence suggests that TAU’s mood effects are less robust than those of CAF, and its influence may depend on interactions with other compounds.

## 4. Discussion

This systematic review was designed to address two distinct objectives: (1) to evaluate the cognitive effects of SCAA restoration in the context of dietary depletion, and (2) to determine the acute effects of supplementation on cognition and mood. Critically, regarding the first objective, no eligible trials were identified, leaving the specific risks of SCAA depletion in plant-based transitions currently untested. Consequently, the findings discussed here pertain exclusively to the second objective—acute supplementation. Within this literature, taurine (TAU) was the only SCAA represented, meaning the effects of methionine (MET) and cysteine (CYS) remain undefined. Overall, the findings present a complex and inconsistent picture of the effects of TAU supplementation on cognition and mood. While few studies explored TAU in isolation, where this was implemented, TAU elicited domain-specific effects rather than global improvement. Interestingly, enhancements in cognitive performance and mood were often more pronounced when TAU was administered in combination with additional active ingredients, such as caffeine (CAF) or glucose. However, the findings surrounding combination interventions were also inconsistent, suggesting that enhancements associated with TAU may be context-dependent, with effects influenced by task demands and ingredient synergy.

In the domain of attention, TAU alone did not enhance attentional performance. However, when combined with glucose [38], or with CAF and glucuronolactone [36], enhancements in attentional control, orienting, sustained attention, and selective attention were observed. The findings suggest that TAU’s effects may be contingent on synergistic interactions between ingredients. Evidence for TAU’s effects on executive function was also largely heterogeneous. One study reported enhanced inhibitory control following TAU supplementation [42], while other studies did not replicate these effects [39,43]. Moreover, although CAF and TAU in combination appeared to enhance cognitive control [42], other findings contrasted this [39,43]. Such discrepancies likely reflect methodological variation, including differences in sample characteristics, cognitive paradigms, and the composition of active interventions.

Findings relating to working memory were similarly heterogeneous and context dependent. Only one study found TAU to enhance working memory capabilities when cognitive demands are high [38], whereas García et al. [41] did not support this finding and reported a possible antagonistic interaction when combined with CAF and glucose, where performance diminished. Reaction time effects were the most inconsistent: in some paradigms, TAU alone produced no significant effects [40,43], while in others it enhanced reaction speed under cognitively demanding conditions [38]. Combinations with CAF produced clearer benefits, particularly during exercise or stress, but in several studies, CAF alone accounted for the improvements. Collectively, the literature indicates that TAU supplementation does not consistently enhance cognition in isolation, with observed effects more plausibly explained by interactions with co-administered bioactive compounds or by task-specific contextual factors.

Since the completion of our searches, Cao et al. [30] have published a systematic review and meta-analysis of RCTs assessing TAU supplementation and cognitive scores in cognitively impaired and non-impaired populations. Across seven RCTs (n = 402), TAU administered alone or alongside exercise training showed no significant overall effect on cognitive scores, and TAU combined with therapeutic drugs did not outperform drugs alone overall; however, subgroup analysis suggested a significant improvement in the Mini-mental state examination when TAU was used adjunctively with therapeutic drugs. These findings are broadly consistent with the present review in that they do not support a robust, generalisable cognitive enhancing effect of TAU. Importantly, Cao et al. [30] primarily synthesised longer-duration, parallel-group trials in primarily cognitively impaired older adults, whereas the current review focuses on acute single-dose trials in healthy adults assessing specific cognitive domains and mood. Together, the two reviews suggest that evidence for TAU cognitive benefits remain limited across both acute performance paradigms and longer-term global cognition in cognitively impaired individuals.

Mood-related outcomes were largely assessed in combination studies, limiting firm conclusions about TAU’s independent contribution. Across trials, TAU with CAF and glucose was associated with increased alertness [37] and reduced state anxiety [41]. When TAU was combined with CAF and glucuronolactone, cognitive performance and mood were significantly improved [36]. However, in the only study to assess TAU alone [38], supplementation influenced ratings of fatigue and lowered ratings of vigour. Divergent outcomes likely reflect variations in study design, intervention duration, and mood assessment sensitivity. Although TAU-containing formulations may modulate mood, evidence does not support a consistent independent effect. Therefore, further controlled trials isolating TAU’s contribution are warranted.

The inconsistent findings may also be partially explained by the neurobiological pathways through which TAU influences psychological outcomes. By modulating inhibitory neurotransmitter systems, such as GABAergic and glycinergic pathways [44], TAU may influence processes such as arousal, attention, and inhibitory control, particularly under conditions of high cognitive load and stress. Importantly, TAU acts on these systems in a dose-dependent manner. Lower concentrations of TAU preferentially activate glycinergic pathways, whereas higher concentrations activate GABA-A receptors [32]. Context-dependent factors such as variations in dose, receptor activation, and task demand may explain heterogeneous effects. Furthermore, these rapid, transient changes in neurotransmission likely represent a distinct pharmacological mechanism from the slower, restorative processes required to address chronic SCAA depletion.

On the other hand, the lack of consistent findings may also be attributed to the influence of TAU on cognition occurring through slower-acting, longer-term mechanisms rather than rapid, transient changes associated with acute supplementation. For example, the human body is relatively inefficient in disposing of an accumulation of unstable molecules known as free radicals [45], which, when abundant, can lead to oxidative stress. Oxidative stress has been associated with disorders of the central nervous system [46]. Due to its reported antioxidant actions in preclinical models (as summarised in Menzie et al. [47]), taurine may help mitigate oxidative stress, suggesting a potential protective role in brain health over time. However, oxidative stress biomarkers were not assessed in the acute randomised controlled trials included in this review, so mechanistic conclusions cannot be drawn. Future research should therefore test longer-term taurine supplementation with mechanistic endpoints, particularly in groups where taurine status may be lower (for example, older adults), who may be more vulnerable to chronic insufficiency of sulphur-containing amino acids [29].

When the cognitive findings are considered in relation to the conditions under which they were generated, it becomes clear that any apparent effects of TAU are highly context-dependent and should be interpreted with caution. Several trials were conducted in athletic populations during, or immediately after, intense exercise bouts. Practically speaking, exercise functions as a powerful contextual modifier: it alters cerebral blood flow, arousal, fatigue, and motivation, and triggers hormonal and catecholaminergic responses (adrenaline, noradrenaline, dopamine) that are themselves capable of changing cognitive performance [48,49,50,51]. These co-occurring changes create distinct context–mechanism–outcome configurations in which TAU is only one element of a much broader physiological state. The type, intensity, and timing of exercise relative to testing further shape these configurations, producing substantial heterogeneity both within and between studies and limiting the extent to which ‘the same’ cognitive domains are truly comparable across trials. Under such conditions, it is difficult to disentangle any independent contribution of TAU from the consequences of the exercise context itself, and any observed benefits are better viewed as arising from specific combinations of context, mechanisms, and tasks, rather than as generalisable drug-like effects of TAU.

Beyond the internal validity of the acute supplementation trials, these findings must be interpreted against a broader nutritional and sustainability backdrop. Many national and international dietary guidelines now encourage shifts towards more plant-based, environmentally sustainable eating patterns. However, if such transitions are not carefully planned and supported, they may contribute to a nutritional “green-gap” [52] where reduced intake of animal-sourced foods could lower intakes of TAU and other sulphur-containing amino acids, for which animal foods are typically the main dietary sources [14,15]. Recent expert group guidelines on nutrition and brain health trials also emphasise the importance of characterising background diet and baseline nutrient status when evaluating cognitive effects of specific compounds [53,54,55,56], and the present literature provides a clear illustration of the limitations that arise when this is not performed. However, none of the trials in this review assessed habitual diet or baseline SCAA status or explicitly recruited individuals with low animal-source food intake (Table 3). Although sulphur amino acid restriction paradigms have been explored in both animals and humans, these studies have primarily targeted metabolic health and ageing outcomes, with cognitive endpoints assessed only in selected rodent models under MET restriction and not in human trials, and none have specifically examined cognitive vulnerability during real-world plant-based dietary transitions [57,58,59,60,61]. Notably, in rodents, MET or sulphur amino acid restriction consistently reduces adiposity, improves insulin sensitivity and lipid profiles, enhances antioxidant defences, and extends lifespan, effects that appear to be mediated by hormonal and transcriptional adaptations such as increased FGF21 signalling [57,58,59]. Emerging human trials in adults with overweight and obesity indicate that whole-food sulphur amino acid restriction is feasible and can produce modest reductions in fat mass alongside characteristic changes in circulating sulphur metabolites and improvements in selected cardiometabolic risk markers [60,61]. However, these studies have not included cognitive outcomes, and none have modelled plant-based dietary transitions in otherwise healthy populations. As a result, the central question motivating this review—whether lower SCAA intake during plant-based transitions confers cognitive vulnerability that can be mitigated by dietary restoration or supplementation—remains unanswered. Future work will need to move beyond short-term, single-dose paradigms in assumed replete samples and instead focus on longer-term interventions in populations with lower SCAA intakes, incorporating careful assessment of habitual diet, biomarkers, and cognitive outcomes. Such studies are essential if we are to understand how to support cognitive health while progressing towards more sustainable dietary patterns.

This systematic review has several methodological strengths. We implemented a rigorous and transparent protocol, preregistered on PROSPERO, and conducted a comprehensive search across multiple major databases in line with PRISMA guidelines. To maximise internal validity, we restricted inclusion to randomised controlled trials and excluded non-randomised or observational designs. Study screening and selection were undertaken independently by two reviewers, minimising selection bias, and all included trials were subjected to duplicate risk-of-bias assessment, further strengthening the reliability of our conclusions.

Despite these methodological advantages, the conclusions should be interpreted while considering several limitations. Firstly, there was substantial heterogeneity among the included studies. While the reviewed evidence and proposed mechanisms suggest the potential for TAU to influence psychological function, it is difficult to draw definitive conclusions regarding TAU’s effects on psychological outcomes from the available data. Moreover, most studies implemented single-dose or short-term interventions using TAU doses exceeding typical dietary intake (estimated at 40–400 mg [31]). This raises concerns about ecological validity and highlights the need for longitudinal studies examining TAU supplementation under more naturalistic conditions. In particular, future work should embed TAU interventions within habitual dietary patterns, including plant-based and plant-forward diets where TAU exposure from animal-sourced foods is lower, to clarify whether any benefits observed at pharmacological doses translate to real-world eating patterns. Longitudinal investigation would better determine the potential cumulative effects of TAU on cognition and mood and more closely replicate human consumption of TAU through diet. In addition, the findings may have limited generalisability as important subgroups, such as older adults, were underrepresented. Given that endogenous TAU concentrations available in the body and brain change across the lifespan [29], further research is warranted in diverse demographic groups, including adolescents [62] and older adults [29], who may be particularly susceptible to deficits in TAU and benefit from supplementation. Finally, to maintain a focus on high-quality evidence from randomised controlled trials, we restricted inclusion to peer-reviewed publications and excluded grey literature.

## 5. Conclusions

Overall, while sulphur-containing amino acids have plausible neurobiological pathways for influencing cognition and mood, the current human evidence from acute supplementation trials is inconsistent and difficult to interpret because of substantial heterogeneity in tasks, populations, and frequent co-supplementation. Importantly, the eligible acute trials were dominated by taurine, with little to no acute randomised evidence isolating methionine or cysteine, so broader claims about “sulphur-containing amino acids” remain underpowered by the existing literature. Consequently, acute taurine supplementation cannot currently be recommended as a reliable cognitive or mood enhancer. However, the broader relevance of SCAAs remains critical. As global food systems transition towards plant-dominant patterns, there is a plausible risk of a nutritional “green-gap” in nutrients that are more concentrated in animal-sourced foods, including taurine, unless transitions are actively supported through appropriate substitution strategies. Future research should therefore move beyond single-dose performance paradigms and test longer-term approaches (dietary planning, supplementation, or both) in well-powered trials, particularly in groups likely to have lower taurine exposure or status and greater vulnerability to dietary shortfalls (for example, older adults and low consumers of animal-sourced foods). Critically, these studies should measure baseline diet and relevant biomarkers to determine whether restoring sulphur amino acid status changes cognitive or mood outcomes, and to ensure sustainability transitions support, rather than inadvertently compromise, cognitive health.

## Figures and Tables

**Table 1:** Inclusion and exclusion criteria applied during study selection.

	Inclusion	Exclusion
Population	Studies involving healthy adults aged 18 years and over were eligible. No restrictions were placed on gender, ethnicity, or geographical location.	Animal studies, as well as those involving children or participants with neurodegenerative diseases or metabolic disorders, were excluded to maintain relevance to the primary population of interest.
Intervention	Supplementation of one or more SCAAs (e.g., taurine (TAU), methionine (MET), or cysteine (CYS)), administered alone or in combination with up to four ingredients (e.g., caffeine or glucose).	Studies using over four total ingredients in the intervention, or those including medications as comparators, were excluded to avoid confounding effects.
Comparison	Must include a placebo control group.	Does not include a placebo control group.
Outcomes	Studies must include outcomes related to cognition (e.g., memory, attention, reaction time, executive function) and/or mood (e.g., anxiety, depression, emotional well-being), measured through validated scales or cognitive tests.	Studies which did not focus on facets of cognitive function and/or mood as outcomes.
Context	Studies conducted in contexts of either of the following: 1. Dietary Depletion/Restoration (e.g., low habitual SCAA intake, plant-based diet transitions). 2. Acute Supplementation/Enhancement (e.g., supradietary dosing in replete individuals).	Contexts involving clinical malnutrition, disease-related deficiency, or recovery from surgery/trauma.
Study type	Peer-reviewed, double-blind, placebo-controlled RCTs.	Non-peer-reviewed, non-randomised trials, cohort or case–control studies, observational designs, and any studies lacking a placebo control were excluded.
Publication Language	English language.	Not published in the English language.

**Table 2:** Cochrane Risk of Bias 2 assessment for randomisation trials.

Study	Randomisation Process	Deviations from Intended Interventions	Missing Outcome Data	Measurement of the Outcome	Selection of the Reported Result	Overall Bias
Seidl et al. [36]	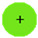	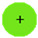	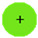	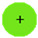	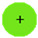	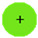
Alford et al. [37]	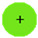	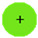	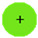	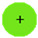	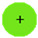	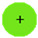
Giles et al. [38]	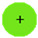	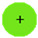	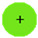	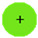	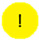	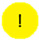
Lassiter et al. [39]	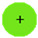	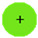	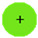	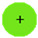	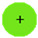	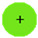
Peacock et al. [40]	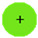	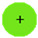	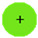	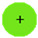	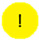	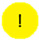
García et al. [41]	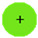	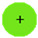	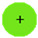	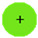	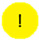	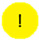
Ozan et al. [42]	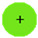	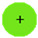	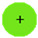	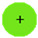	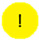	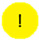
Liu and Rong [43]	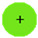	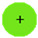	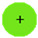	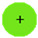	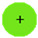	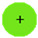

Note: ‘+’ = low risk of bias, and ‘!’ = some concerns of bias.

**Table 3:** Characteristics of included studies, including population, aims, design, intervention (with dosages), outcome measures (with cognitive tests), mood measures, main findings, and authors’ conclusions.

Reference	Population	Aims	Design	Intervention and Dosage	Cognitive Tests	Mood Measures	Main Findings	Habitual Diet/SCAA Status Assessed?	Authors’ Conclusions
Seidl et al. [36]	N = 10 graduate students (23.9 years old ± 2.5) 6 female, 4 male Regular CAF consumers *n* = 5 Non-CAF consumers *n* = 5 All healthy, non-smokers	To evaluate the combined effects of CAF **, TAU *, and glucuronolactone (CTG) on cognitive performance and mood (replicated quantities of Red Bull drink). To test whether cognitive and mood effects of these ingredients occur at night, when participants are expected to be more fatigued.	Double-blind, placebo-controlled, crossover, repeated-measures design. Two test sessions separated by at least one week. Participants were randomly assigned to receive either CTG or PLA *** (wheat-bran capsules) and then switched for the other session. All participants had abstained from CAF and alcohol for at least 24 h before the test.	Single dose: 1 g TAU, 80 mg CAF, +600 mg glucuronolactone across 7 capsules Capsules taken with 250 mL water	D2 test of attention P300 ERP wave	Basler Befindlichkeitsbogen Questionnaire	CAF, TAU, and glucuronolactone combination improved RT and D2 attention scores vs. PLA. P300 latency was slowed in PLA. The CAF, TAU, and glucuronolactone group showed non-significant shorter P300 latencies in comparison with pretreatment. PLA experienced significant declines in well-being, vitality, and social extroversion by the end of the session. The active intervention group did not show this decline.	No—CAF-user vs. non-user status recorded; no broader habitual diet or SCAA measures	The combination of CAF, TAU, and glucuronolactone (CTG) significantly improves cognitive performance and mood, especially during periods of fatigue (late night). These effects are not merely due to reversing CAF withdrawal, as non-CAF users benefited similarly to CAF users.
Alford et al. [37]	3 studies Study 1: N = 10 (5 f, 5 m) aged 18–30. Study 2: N = 14 (7 f, 7 m) aged 18–35 Study 3: N = 12 (5 f, 7 m) aged 20–21 Total: *N* = 36 (17 f, 19 m) aged 18–35; healthy, moderate CAF users	To investigate the effects of Red Bull energy drink on physical endurance (aerobic and anaerobic), psychomotor performance (reaction time, concentration, memory), subjective alertness, and mood.	Double-blind, repeated-measures, randomised crossover across three separate studies. Each study completed within a four-week period with a one-week break for each participant between their two testing sessions. Participants received Red Bull, water (still or carbonated), or a PLA (still water replaced carbonated water in the 3rd study).	All studies used a single-dose combination of Red Bull (250 mL) which included TAU 1 g, CAF 80 mg, glucose 5.25 g, and glucuronolactone 600 mg Study 1 PLA = carbonated water Study 2 PLA = carbonated water OR no drink control Study 3 PLA = Still water OR ‘Dummy Energy drink’ (flavoured carbonated water)	5-choice reaction time ‘Concentration Task’ Immediate recall Memory task	VAS scale—100 mm	Red Bull significantly improved choice reaction time, concentration, and immediate recall compared to control drinks and PLA. Participants reported increased alertness after consuming Red Bull compared to PLA and control drinks.	No—only caffeine-use status (moderate CAF users) recorded; no assessment of habitual diet or SCAA intake	Red Bull energy drink improves both mental and physical performance, including reaction time, memory, concentration, and endurance. These effects are attributed to the combined ingredients CAF, TAU, and glucose. The drink also increases subjective alertness without significant cardiovascular side effects at rest. The authors commend TAU for ‘other’ positive effects on mood.
Giles et al. [38]	N = 48 habitual CAF consumers, 18 M, 30 F Good health, CAF consumers (200 mg/day+), non-smokers, no use of prescription medication except for oral contraceptives	To evaluate the individual and combined effects of CAF, TAU, and glucose on cognitive performance and mood in habitual CAF consumers who were CAF-deprived for 24 h.	Double-blind mixed design Within subject, 4 conditions, 3-day washout: Within-participants factors: CAF and TAU treatment. Between-participants factor: glucose treatment or PLA.	Single-dose, combined, and PLA separated by a 3-day washout: PLA = 0 CAF + 0 TAU TAU = 0 CAF + 2000 mg TAU CAF = 200 mg CAF + 0 mg TAU CAF × TAU = 200 mg CAF + 2000 mg TAU Between-group factor: Half participants administered 50 g glucose (250 mL GLU + sparkling water) or PLA (250 mL sparkling water + 250 mL PLA)	Attention (alerting, orienting, executive control), reaction time, working memory, and psychomotor performance Attention Network Test (ANT) N-back task Reaction time task (RTT)	Mood states, CAF withdrawal symptoms Profile of Mood States (POMS) Withdrawal Questionnaire (WQ)	TAU increased choice reaction time accuracy and improved reaction times in particular working memory tasks (verbal and object N-back). TAU + GLU increased orienting attention. Glucose improved object working memory in combination with CAF. CAF improved executive control and working memory, and reduced reaction times. It also increased tension and vigour, and reduced fatigue and withdrawal symptoms.	No—caffeine consumption used as inclusion criterion; no assessment of habitual diet or SCAA intake	TAU had inconsistent effects on mood and cognitive performance. CAF was the main driver of cognitive performance improvements, particularly in attention, working memory, and psychomotor performance. Glucose had limited effects on cognitive performance, and its interaction with CAF and TAU requires further research.
Lassiter et al. [39]	N = 15 healthy trained cyclists (7 f, 8 m), aged 20–45 years	To evaluate the effect of an ED containing CAF, carbohydrates, TAU, and Panax ginseng on cycling time-trial performance and cognitive performance at rest, during exercise, and after exercise.	Double-blind, placebo-controlled, randomised, crossover repeated-measures. Each participant completed two experimental trials separated by 6–21 days. Participants consumed either the ED or a PLA following a 12 h fast and CAF abstention followed by a 35 km cycling time-trial course after intervention.	Single-dose energy drink intervention: 480 mL containing 54 g carbohydrates, 160 mg CAF, 2 g TAU, 400 mg Panax ginseng PLA = 480 mL 0 kcal, CAF-free, no herbal or amino acids	Choice reaction time task Go/no-go task (executive function) Stroop Test Tapping task—taps per second psychomotor control test	N/a	Improved performance was observed on the executive function task and reduced movement times after the race in both the choice reaction and executive function tasks; this was a time effect. PLA also improved post-race. Stroop test reaction times improved post-race but showed no significant treatment effects. Energy drink intervention increased taps per second in the tapping task both pre- and post-exercise compared to PLA.	No—trained athlete status reported; no habitual diet or SCAA intake assessed	The energy drink enhanced both aerobic performance and certain aspects of cognitive function (tapping speed, executive function) during and after exercise.
Peacock et al. [40]	N = 19 right-handed females, 19–22 years old (M = 20.8)	To investigate the independent and combined effects of CAF and TAU on behavioural performance, specifically reaction time.	Double-blind, placebo-controlled, crossover design. Four counterbalanced conditions: PLA, TAU, CAF, CAF × TAU. Participants’ sessions were separated by a 2–7-day washout period.	Single-dose, combined, and PLA separated by 2–7-day washout TAU = 1 g CAF = 80 mg PLA = matched to active counterparts’ weight with cornflour	Reaction times (visual oddball task) Stimulus degradation task: Measures reaction time to identify digits at three levels of visual degradation (intact, low degradation, high degradation)	N/a	Non-significant effects of TAU for reaction times in either task. No significant effects of CAF on visual oddball task. CAF significantly improved reaction times in the stimulus degradation task compared to PLA. CAF × TAU did not enhance reaction times compared to CAF. TAU may attenuate CAF’s beneficial effects on reaction time.	No—no habitual diet or SCAA intake reported	Treatments are task-dependent. TAU did not have significant independent effects on reaction time but may attenuate CAF’s performance-enhancing effects in particular tasks. The interaction between CAF and TAU requires further research to understand its impact on performance outcomes.
García et al. [41]	N = 80 healthy medical students (50 m, 30 f), mean age 21.45. All participants had consumed energy drinks in their lifetime	To determine the acute effects of different energy drinks on cardiovascular parameters, stress levels, and working memory in medical students.	Double-blinded, randomised placebo-controlled trial. Four groups: control group (carbonated water); groups A, B, and C were commercially available energy drinks. Tests were conducted before and after consumption of intervention.	Single-dose energy drink intervention: All drinks 460 mL A: CAF = 149.5 mg, glucose = 23 g, TAU = 0 g B: CAF = 147.2 mg, glucose = 49.6 g, TAU = 1.84 g C: CAF = 155 mg, glucose = 52.8 g, TAU = 1.95 g Control: carbonated water	N-back task	State–Trait Anxiety Inventory (STAI)	Group A showed an increase in working memory performance (no TAU) compared to the control, but no significant differences between groups were found. The STAI test showed a decrease in anxiety in group C.	No—prior energy drink exposure noted; no systematic assessment of habitual diet, TAU, or SCAA intake	The results highlight the variability of energy drink effects on physiological and cognitive functions, likely due to differing compositions of the drinks. The authors suggest anxiety reduction could be due to ingredient composition.
Ozan et al. [42]	N = 20 male, elite boxers (>10 years’ experience) 18–24 years old (M = 22.14 ± 1.42)	To evaluate the effects of CAF, TAU, and their combination CAF × TAU compared to PLA on athletic performance and exercise-induced fatigue cognitive performance levels.	Double-blind randomised crossover. Four conditions: CAF, TAU, CAF × TAU, PLA. All conditions met by each participant in a 72-h period.	Single-dose, combination, and PLA within 72 h window TAU = 3 g CAF = 6 mg/kg CAF × TAU = 6 mg/kg + 3 g PLA = 300 mg of maltodextrin	Reaction times and accuracy Stroop test	N/a	CAF × TAU improved cognitive reaction times and accuracy compared to PLA. TAU significantly improved incongruent Stroop trial accuracy and incongruent trial reaction times vs. PLA.	No—no habitual diet or SCAA intake reported	Co-ingesting CAF and TAU improved anaerobic performance, balance, agility, and cognitive function in elite male boxers more effectively than either a supplement alone or PLA.
Liu and Rong [43]	N = 16 healthy male university footballers (mean 23.7 y)	To assess acute effects of TAU, CAF, and TAU + CAF on cognition (Stroop) and exercise performance under hypoxia.	Double-blind randomised placebo-controlled crossover RCT. Four groups: PLA, CAF, TAU, TAU × CAF. Tests were conducted 60 min after ingestion; 3-day washout period.	Single-dose and combination, 3-day washout CAF = 5 mg/kg TAU = 50 mg/kg CAF × TAU = 50 + 5 mg/kg PLA = maltodextrin 5 mg/kg Stroop was administered after a physical warm-up (BL), after an exhaustion test (MID), and after intense sprinting (END)	Stroop task	N/a	CAF improved reaction time vs. PLA for congruent and incongruent Stroop trials; no change in accuracy. TAU alone showed no significant results. For incongruent and congruent trials, CAF caused significantly faster RT than either TAU and PLA. For incongruent trials, CAF improved RT vs. TAU × CAF.	No—no habitual diet or SCAA intake reported	CAF is the primary driver of cognitive enhancement in Stroop task performance. TAU alone or with CAF is ineffective and did not enhance performance during the Stroop task.

* TAU = taurine, ** CAF = caffeine, *** PLA = placebo.

## Data Availability

The data presented in this study are available in Preregistration and data extraction are available via Prospero: CRD42024574453. These data were derived from the following resources available in the public domain: https://www.crd.york.ac.uk/PROSPERO/view/CRD42024574453 [accessed on 13 January 2026].

## Supplementary Materials

The following supporting information can be downloaded at https://www.mdpi.com/article/10.3390/foods15040634/s1: Table S1: PRISMA 2020 Checklist.

## Abbreviations

The following abbreviations are used in this manuscript:

CAF	Caffeine
PLA	Placebo
SCAA	Sulphur-containing amino acid
TAU	Taurine
